# Microalbuminuria among Type 1 and Type 2 diabetic patients of African origin in Dar Es Salaam, Tanzania

**DOI:** 10.1186/1471-2369-8-2

**Published:** 2007-01-15

**Authors:** Janet Joy Kachuchuru Lutale, Hrafnkell Thordarson, Zulfiqarali Gulam Abbas, Kåre Vetvik

**Affiliations:** 1 Institute of Medicine, Division of Haraldsplass Deaconal Hospital, University of Bergen, Bergen, Norway; 2 Centre for International Health, University of Bergen, Bergen, Norway; 3 Muhimbili University College of Health Sciences, Dar es Salaam, Tanzania; 4 Department of Medicine, Haraldsplass Deaconal Hospital, Bergen, Norway; 5 Department of Medicine, Haukeland University Hospital, Bergen, Norway; 6 Abbas Medical Centre, Dar es Salaam, Tanzania

## Abstract

**Background:**

The prevalences and risk factors of microalbuminuria are not full described among black African diabetic patients. This study aimed at determining the prevalence of microalbuminuria among African diabetes patients in Dar es Salaam, Tanzania, and relate to socio-demographic features as well as clinical parameters.

**Methods:**

Cross sectional study on 91 Type 1 and 153 Type 2 diabetic patients. Two overnight urine samples per patient were analysed. Albumin concentration was measured by an automated immunoturbidity assay. Average albumin excretion rate (AER) was used and were categorised as normalbuminuria (AER < 20 ug/min), microalbuminuria (AER 20–200 ug/min), and macroalbuminuria (AER > 200 ug/min). Information obtained also included age, diabetes duration, sex, body mass index, blood pressure, serum total cholesterol, high-density and low-density lipoprotein cholesterol, triglycerides, serum creatinine, and glycated hemoglobin A_1c_.

**Results:**

Overall prevalence of microalbuminuria was 10.7% and macroalbuminuria 4.9%. In Type 1 patients microalbuminuria was 12% and macroalbuminuria 1%. Among Type 2 patients, 9.8% had microalbuminuria, and 7.2% had macroalbuminuria. Type 2 patients with abnormal albumin excretion rate had significantly longer diabetes duration 7.5 (0.2–24 yrs) than those with normal albumin excretion rate 3 (0–25 yrs), p < 0.001. Systolic and diastolic blood pressure among Type 2 patients with abnormal albumin excretion rate were significantly higher than in those with normal albumin excretion rate, (p < 0.001).

No significant differences in body mass index, glycaemic control, and cholesterol levels was found among patients with normal compared with those with elevated albumin excretion rate either in Type 1 or Type 2 patients.

A stepwise multiple linear regression analysis among Type 2 patients, revealed AER (natural log AER) as the dependent variable to be predicted by [odds ratio (95% confidence interval)] diabetes duration 0.090 (0.049, 0.131), p < 0.0001, systolic blood pressure 0.012 (0.003–0.021), p < 0.010 and serum creatinine 0.021 (0.012, 0.030).

**Conclusion:**

The prevalence of micro and macroalbuminuria is higher among African Type 1 patients with relatively short diabetes duration compared with prevalences among Caucasians. In Type 2 patients, the prevalence is in accordance with findings in Caucasians. The present study detects, however, a much lower prevalence than previously demonstrated in studies from sub-Saharan Africa. Abnormal AER was significantly related to diabetes duration and systolic blood pressure.

## Background

Diabetic nephropathy accounts for a significant reduction in life expectancy of diabetic patients. It is the leading cause of end-stage renal disease in the western world [1,2]. In Caucasians, the cumulative incidence of diabetic nephropathy in Type 1 and Type 2 diabetic patients has been estimated to range from 20% to 27% after a diabetes duration of 20 years [3,4].

Extensive studies in the Western world have demonstrated that diabetic patients with microalbuminuria have increased risk of progression to overt proteinuria, and after some time, renal failure. The progression of diabetic nephropathy from the appearance of clinical proteinuria to end stage renal failure is usually irreversible. Without any intervention, approximately 80% Type 1 patients with persistent microalbuminuria develop overt nephropathy after 10–15 years. Eventually 50% of these develop end stage renal failure within 10 years and 75% by 20 years [5]. In Type 2 diabetic patients, 20–40% with microalbuminuria progress to overt nephropathy and 20 years later, approximately 20% develop end stage renal failure [6].

There is a racial difference in the prevalence of diabetic nephropathy and end stage renal failure. African American patients for instance have been reported to suffer greater diabetic nephropathy and kidney damage than Caucasian Americans [7,8].

Information on nephropathy in African diabetic populations is scarce. Available data between 1971 and 2002 shows the prevalence of microalbuminuria in Africa to vary between 26% and 57% in diabetic patients, with variable duration of the disease [9-12], [Table 1]

**Table 1:** Prevalences of microalbuminuria in people with diabetes among Africans

**Study**	**Type of diabetes**	**N**	**Age (years)**	**Duration (years)**	**urine sample**	**AER criteria**	**albumin assay**	**Prevalences**
Erasmus, RT 1992 Nigeria	Type 2	113	51.1 yrs	0.2–25	24 hr urine one sample	Micro = ≥30 mg/24 hr	Immunodiffusion	microalb 57% 52%-dur<5 yrs
Rahlenbeck, SI 1997 Ethiopia	IDDM-	71	32.6 ± 9.5 30.6 ± 8.9	6.0 ± 4.9 11.4 ± 6.7	Spot morning (3 consecutive visits)	Urinary alb conc. micro = 30–299 mg/L macro = >300 mg/L	MICRAL	microalb-32% macrolb-15%
	NIDDM-	99	55.4 ± 13 59.9 ± 8	5.3 ± 3.9 10.3 ± 5.5				microalb-37% macroalb-20%
Sobngwi, E 1999 Cameroon	Type 1 Type 2	9 55	19–70 yrs	1–23 yrs	Overnight urine	UAE rate micro = 30 mg/24 hr	MICRAL	microalb 53,1%
Wanjohi, FW 2002 Kenya	Type 2 New	100	53.7 ± 9.3	10.3 ± 7.5 (months)	Spot morning-one sample	Urinary alb conc. Cut of limits not defined	MICRAL	microalb-26% macroal b-1pt
Current Study 2006 Tanzania	Type 1 Type 2	91 153	21 (4–44.8) 53 (24–85)	3(0–17) 4(0–25)	Timed overnight 2 samples	AER	Immunoturbidimetry	T1micro-12.1% T1macro-1.1% T2micro-9.8% T2macro-7.2%

Early medical treatment and lifestyle adjustments have been shown to halt the progression from micro- to macroalbuminuria and eventually end stage renal failure [13,14]. Therefore, detection of microalbuminuria as early as possible in the course of the disease is very important. In the developing countries, this is even more so because of the economical constraints, kidney replacement therapy is seldom an option.

The aim of this study was to determine the prevalence of microalbuminuria and diabetic nephropathy among Type 1 and Type 2 African diabetic patients in Dar es Salaam. We also wanted to assess the interrelation of microalbuminuria with basic patient socio-demographic features as well as clinical and biochemical parameters.

## Methods

Patients attending the Muhimbili outpatient diabetic clinic for their regular visits and who accepted to participate in the study were included. The study was done from July 2003 to March 2004. Informed consent verbally and in writing was obtained from all patients and were interviewed using a standardized questionnaire. The history including date of diabetes diagnosis, family history of diabetes, history of cigarette smoking, age, gender and date of presentation were obtained.

Patients were enrolled in two steps. In step 1, 204 consecutive patients were included. Secondly, to enlarge the group of Type 1 patients, 68 consecutive patients aged 30 years or younger and on insulin therapy were enrolled. Patients were classified into Type 1 and Type 2 according to the WHO clinical stage criteria [15] using information from each patient's diabetes sheet and data collected at enrolment. Patients not fitting into the clinical features of either type were classified as undetermined.

### Clinical assessment

Weight was measured without shoes or heavy clothing with a SECCA^® ^scale. It was recorded to the nearest 0.5 kilogram. Height was determined to the nearest 0.5 cm with a rigid measure against a vertical wall.

Blood pressure was measured with a suitable mercury sphygmomanometer. After a 10 minutes rest with the patient in the sitting position, blood pressure was measured two times at 5 minutes interval. The 1^st ^and the 5^th ^Korotokoff's sound were used to determine the systolic (SBP) and diastolic blood pressure (DBP) measurements respectively. The second blood pressure measurement was used as the blood pressure for the individual. Hypertension was defined as SBP ≥140 and/or DBP of ≥90 mmHg or use of hypertensive medication [16].

### Blood sample collection and storage

A morning sample of 6–9 mls of venous blood was drawn from each subject and centrifuged within 2 hours of collection. The sera and urine samples were frozen at -80°C and transported to Bergen using a dry ice-filled container.

Capillary blood glucose was measured using a HemoCue AB glucose analyzer (Angelholm, Sweden). HbA_1_c was measured immunochemically at the clinic using DCA 2000^®^+ (Bayer Corporation) [17]. The instrument is standardized against the DCCT method [18]. Quality was controlled using standard solutions.

### Biochemical assays

Assessment of serum potassium, sodium, serum cholesterol, high-density lipoproteins, and low-density lipoprotein, serum triglycerides, serum creatinine were done at the Haukeland University hospital, Norway. Biochemical tests were analyzed on a BM/Hitachi 917 Auto Analyzer. (Boehringer Diagnostics, Mannheim) using kits supplied by Boehringer Mannheim (Mannheim, Germany). Serum and urine creatinine measured by modified kinetic Jaffé reaction, serum cholesterol by Cholesterol Oxidase p-aminophenazone (CHOD-PAP) and serum triglycerides by Glycerol phosphate oxidase p-aminophenazone (GPO-PAP) methods.

### Urine collection

Two overnight urine specimens were collected on consecutive nights. All patients received clear oral and written instruction on how to collect a timed overnight urine sample in a 2-liter plastic container with no preservatives. Patients were instructed to empty the bladder and discard the urine just before going to bed and to note the time. All the urine collected after going to bed (sleeping time) and first morning urine sample on waking up was collected into the container and the time of the first morning sample recorded. Sample were delivered to the clinic the same morning. Patients who were unable to comply with the instructions or lived far from the hospital were admitted and samples collected in the hospital. Collected urine were measured and screened for signs of infection using a dipstick (Multistix^® ^10SG reagent strips, Bayer Diagnostics, UK) and microscopically. Samples found to be infected were discarded; the patient treated accordingly and after a week of cure allowed to re-submit another sample. Samples were then frozen at -80°C until analysis for albuminuria and urine creatinine in Norway.

### Assessment of urinary albumin excretion

Urine albumin concentrations were determined using an automated immunoturbidity method, (anti-human serum albumin obtained from Dako, Oslo) with sensitivity of 2.3 mg/L and inter- and intra assay coefficients of variation of 4.4% and 4.3% respectively (Dade Behring Marburg Gmbh.USA). We used the average of the two readings to determine the final urinary excretion rate (AER). AER was then categorized as normoalbuminuria (AER of < 20 μg/min), microalbuminuria (20–200 μg/min) and macroalbuminuria (> 200 μg/min). Albumin to creatinine ratio (ACR) was calculated and values ≥ 2.5 mg/mmol for men, ≥3.5 mg/mmol for women defined as microalbuminuria. Macroalbuminuria was defined as ACR ≥30 mg/mmol for men and women.

### Renal function

Creatinine clearance calculations using the Cockcroft Gault Formula were used as a correlate of eGFR [19]. Findings were classified according to stages of renal dysfunction as proposed by the National Kidney Foundation [20].

### Ethics

The study was done in accordance with the amended 2000 Helsinki declaration. The Scientific and Publication committee at the Muhimbili University College of Health Sciences (MUCHS) and the Norwegian Regional Medical Research Ethics Committees of Western Norway approved the study protocol.

### Statistical analysis

Distribution within groups was tested for skewness and statistically significant differences of standard deviations. Parametric and nonparametric tests were then used as appropriate. Differences between proportions were assessed by Fisher's Exact test or Chi-square test. Multiple linear regression was performed with ln AER as the dependent variable. Data were analyzed using SPSS version 12 for Windows and GraphPad INSTAT-version 3.00 supplied by the GraphPad Software, Inc San Diego, USA. P < 0.05 was considered statistically significant.

## Results

### Socio-demographic characteristics of the study population

Two hundred seventy one patients were consecutively enrolled, 13 excluded because of incomplete data. Among the remaining 258 patients (54.3% females), 198 were enrolled in step one, 31 (15.6%) classified as Type 1, 153 (77.3%) as Type 2 and 14 (7.1%) as undetermined. Step two recruited 60 Type 1 patients making 91 Type 1 patients. Only data of Type 1 and Type 2 patients were analyzed further.

Type 1 patients were significantly younger than Type 2 patients (median age 21 vs 53 years, p < 0.001), had shorter known duration with diabetes (3 vs 4 years, p < 0.05) and had poorer glycaemic control (median HbA1c 13.9% vs 9.9%, p < 0.001) than Type 2 patients. The median SBP, DBP, serum creatinine and mean BMI levels for Type 1 patients were significantly lower than those of Type 2 patients (p < 0.001), [Table 2].

**Table 2:** Sociodemographic characteristics and basic data of Type 1 and Type 2 diabetic patients

**Variables**	**Type 1 diabetes N = 91**	**Type 2 diabetes N = 153**
Male N (%) ‡‡	45(47.4)	68 (44.2)^ns^
Age at inclusion (yrs) ‡	21(4–44.8)	53 (23.5–85) ***
Diabetes duration (yrs) ‡	3 (0–17)	4 (0–25)*
BMI (kg/m^2^) (mean ± SD) #	19.3 ± 3.8	27.8 ± 4.8***
SBP (mmHg)‡	108 (72–140)	134 (90–210) ****
DBP (mmHg)‡	74 (50–110)	84 (52–136) ****
Serum Creatinine (umol/L) ‡	47 (14–124)	71 (6–234) ***
HbA1c (%) ‡	13.9 (6.3–>14)	9.9(4.4–>14) ***

Data expressed as median (min-max) unless stated otherwise.

P values – * p < 0.05, **p < 0.01, *** p < 0.001, ****p < 0.0001

‡‡ Fisher's Exact test, ‡ Mann-Whitney test, #Unpaired t-test

### Albuminuria

Prevalence among Type 1 patients recruited in phase 1 was 5(16.7%) microalbuminuria, 0% macroalbuminuria and for Type 1 patients recruited in phase 2, 6(9.8%) microalbuminuria and 1(1.6%) macroalbuminuria, making a combined prevalence of 12.1% microalbuminuria and 1.1% of macroalbuminuria i.e. 12 (13.2%) had abnormal AER. The corresponding prevalences for Type 2 patients were 26 (17%) for abnormal AER, 9.8% microalbuminuria and 7.2% macroalbuminuria.

A significant number of Type 2 patients 33 (22%) as compared with 4 (4.6%) of Type 1 patients had reduced glomerular filtration rate (GFR < 60 ml/min), (p < 0.001). No patient was found to have end stage renal failure (GFR < 15 mls/min).

### Albuminuria and the associated risk factors

The median diabetes duration, SBP, DBP and proportion of patients on anti-hypertensive drugs among Type 2 patients with abnormal AER was significantly higher than for those patients with normal AER were (p < 0.001), [Table 3].

**Table 3:** Albuminuria in relation to age, sociodemographic, blood pressure and biochemical data among Type 1 and Type 2 patients

	**Type 1 diabetes N = (91)**		**Type 2 diabetes N = (153)**	
	
	**NORMAL (N = 79) (AER ≤20 ug/min)**	**ABNORMAL (N = 12) (AER > 20 ug/min)**	**NORMAL (N = 127) (AER ≤20 ug/min)**	**ABNORMAL (N = 26) (AER > 20 ug/min)**
Age at inclusion (yrs) ‡	20.0 (4–44.8)	21.5 (11–38)	51.6 (23.5–85)	54(36.6–80)
Duration of diabetes (yrs) ‡	3.0 (0–17)	4.2 (0–9)	3 (0–25)	7.5(o.2–24)***
BMI (kg/m^2^) (mean ± SD) #	19.3 ± 3.7	18.8 ± 4.4	27.9 ± 5	27 ± 4.7
SBP mmHg ‡	109 (72–140)	108 (90–140)	130(90–210)	160 (110–200)***
DBP mmHg ‡	72 (50–110)	80 (54–100)	82(52–120)	92 (70–136)***
AER (ug/min) ‡	4.9 (0.65–19.9)	51 (22.7–290.2)***	4.67 (1.29–18.92)	164.6 (22.1–2000)***
Creatinine clearance (mls/min) ‡	129(46–481)	132(96.4–191)	107(40–273)	86.6(40–178.5)
HbA1c (%) ‡	13.5(6.3–>14)	14(8.9–>14)	9.6(4.4–>14)	10.1(5.4–>14)
Proportion anti HT medication N(%)‡‡	0	0	31(24)	15(58)**
Proportion with Hypertension N(%)‡‡	7(9.7)	2 (18.2)	59(47.6)	21(80.8)**

Data expressed as median (min-max) unless stated otherwise.

‡ Mann Whitney test, # unpaired t test, ‡‡ Fischer's exact test

p value – *p < 0.05, **p < 0.01, ***p < 0.001

The differences in age at inclusion, diabetes duration, SBP, DBP, HbA1c, BMI, and creatinine clearance distribution between patients with normalbuminuria and abnormal AER levels were not statistically significant in Type 1 patients.

Of the 45 Type 2 patients using anti-hypertensive medication, 13 were using ACE-Inhibitors, 12 b-blockers, 7 calcium channel blockers, 5 thiazides and 8 other drugs.

Type 2 patients with known diabetes duration of more than 5 years had significantly larger proportion of patients with abnormal AER levels (p < 0.05), higher DBP (p < 0.05), and poorer glycaemic control (p < 0.001) than those patients with diabetes duration of 5 years or less. No significant differences were noted in all the variables in both duration ranges among Type 1 patients [Table 4].

**Table 4:** Abnormal albuminuria (AER), age at inclusion, metabolic and blood pressure control and serum creatinine levels in relation to duration below and above 5 years among Type 1 and Type 2 diabetes.

	**Type 1 diabetes N = 91**		**Type 2 diabetes N = 153**	
	
	**Duration ≤5 yrs n = 73**	**Duration >5 yrs n = 18**	**Duration ≤5 yrs n = 91**	**Duration >5 yrs n = 62**
Abnormal AER N (%)‡‡	8 (10.9)	4(22.2)	9 (9.9)	17 (27.4)**
Microalbuminuria N (%)	7(9.6)	4(22.2)	8(8.8)	7(11.3)
Age at inclusion (yrs) ‡	20.0 (4–44.8)	24.3(13.6–43)	51.5(23.5–80)	53.8(35.3–85)
Diabetes duration (yrs) ‡	2 (0.0–5.0)	8.7(5.5–17)***	2.0 (0.0–5.0)	9.0 (5.5–25.0)***
BMI kg/m^2 ^(mean ± SD) #	19.3 ± 3.9	19.0 ± 3.3	28.4 ± 5.1	26.8 ± 4.1*
SBP mmHg ‡	102 (72–140)	110 (90–140)	134(90–210)	140 (90–200)
DBP mmHg ‡	70 (50–110)	77 (60–98)	81(52–106)	88 (60–136)*
Creatinine clearance (mls/min) ‡	128 (46–481.8)	137.2 (96–161)	105(40–273.4)	106.4 (40.2–190.6)
Hba1c (%) ‡	14 (7.4–>14)	12.7 (6.3–>14)	8.4 (4.4–>14)	10.8 (5.8–>14)***

Data expressed as median (min-max) unless stated otherwise

**Tests **– ‡‡Fischer's exact test#, ‡Mann Whitney test, # Unpaired t test

**P value **– *p < 0.05, **p < 0.01, ***p < 0.001

Only six subjects (2.4%) were current smokers, 11.5% had stopped smoking and 86.1% had never smoked. All patients who were current smokers had normal albumin excretion rates and only three of those who had stopped smoking had abnormal AER.

### Regression analysis

Linear regression analysis was performed with log(n) AER as the dependent variable and diabetes duration, age at inclusion, gender, BMI, HbA1c, SBP and DBP, serum creatinine and cholesterol as covarites. Among Type 1 diabetic patients a simple linear regression analysis demonstrated a significant association between diabetes duration (p = 0.045). Among Type 2 patients, a simple linear regression analysis revealed diabetes duration (p < 0.001), SBP (p < 0.001), DBP (p = 0.007) and serum creatinine (p < 0.001) to be correlated with log AER [Table 5].

**Table 5:** Linear regression analysis of albuminuria (ln AER) with respect to potential covariates

	***Type 2 diabetes***					
	***Univariate analysis***			Adjusted analysis*		
***Covariates***	***β***	***95% CI***	***p-value***	***β***	***95% CI***	***p-value***

Diabetes duration (yrs)	0.117	0.076, 0.159	0.000	0.090	0.049, 0.131	0.000
Age at inclusion (yrs)	0.003	-0.019, 0.025	ns			
Gender-Male	-0.283	-0.777, 0.211	ns			
BMI (kg/m2)	-0.043	-0.095, 0.010	ns			
HbA1c %	0.065	0.035, 0.164	ns			
SBP (mmHg)	0.019	0.010, 0–029	0.000	0.012	0.003, 0–021	0.010
DBP (mmHg)	0.023	0.006, 0.039	ns			
S. creatinine (mmol/l)	0.021	0.012, 0.030	0.000	0.011	0.002, 0.020	0.016
S. cholesterol (mmol/l)	0.048	-0.149, 0.245	ns			

* Results from forward/backwards stepwise selection Predictors in the model, diabetes duration, SBP, age, serum creatinine

A backward stepwise multiple linear regression analysis by elimination procedure was used to identify factors that might independently be associated with development of albuminuria. Diabetes duration (p < 0.001) was picked as the strong predictor for the development of AER and SBP (p = 0.010) and serum creatinine (p = 0.016) for Type 2 diabetes but no variable for Type 1 diabetes was picked on multiple regression analysis.

Comparison of methods of albuminuria detection using AER as the gold standard measurement and ACR, both using average albumin levels detected on two overnight urine samples using the RIA were made. The calculated ACR showed a sensitivity of 81.6%, specificity of 99% with a kappa coefficiency of agreement of 0.85, (95%CI 0.76, 0.95).

## Discussion

This cross sectional study reveals a prevalence of microalbuminuria and its determinants in a selected population from a diabetic clinic in Dar es Salaam, Tanzania. Prevalence for microalbuminuria (12.1%) among Type 1 patients in this study who had median diabetes duration of 3.0 years was relatively high when compared with prevalences found among Type 1 Caucasians diabetic patients with similar diabetes duration. In a longitudinal study, a cumulative incidence of about 4% was found in 148 Type 1 diabetic patients after diabetes duration of three years [21]. Other studies in Caucasians have reported prevalences of microalbuminuria in Type 1 patients from 7–22% [22-24] however, in these studies disease duration was much longer than in the present study. Among the Type 2 patients in our study microalbuminuria was 9.8%, and median diabetes duration 4 (0–25) years. This is comparable with findings in Caucasian Type 2 patients (8–32%) [22,23,25-27].

This study is one of relatively few from sub-Saharan Africa dealing with microalbuminuria. A Medline search on microalbuminuria in diabetic patients revealed four comparable studies from sub-Saharan Africa [Table 1] which display relatively high prevalences compared with our findings. Screening for bacteriuria was done in these studies too. They used mainly the "MICRAL test" for urinary albumin assessment. The MICRAL test is less sensitive than the immunoturbidimetry method employed in our study. The way of assessing albuminuria in this study should not have caused lower prevalence of microalbuminuria but rather the opposite. Some studies have shown decay in albumin concentration when urine samples were kept frozen at -20°C, but not with storage temperature of -80°C as was applied in this study [28-30]. Even if freezing and thawing should have caused an albumin decay in our urine samples as well, this reduction could hardly account for the big difference in albumin excretion rates demonstrated between the present study and the former studies from sub-Saharan Africa. Differences in design, setting, methods for collection of urine and assessment of microalbuminuria, and patient populations may contribute to the differences found between these African studies.

From table 1, one might get the impression of a decreasing trend in the prevalence of microalbuminuria in Africa. Decreasing prevalences of microalbuminuria and microvascular complications have been reported from western countries, which is attributed to improvement in metabolic control, better treatment of hypertension, the use of angiotensin converting enzyme inhibitors and angiotensin receptor blockers [31,32]. Even though tempting, it is not appropriate to draw any firm conclusion, from the studies listed in table 1, as to whether prevalence of microalbuminuria really is on a decrease in sub-Saharan Africa. This is especially so since the use of angiotensin receptor blockers and converting enzyme inhibitors is limited.

It is uncommon in Caucasian Type 1 patients to diagnose microalbuminuria during the first five year [33,34]. In the study by Marshall et al, only one patient with Type 1 diabetes of less than 5 years duration had an AER in the microalbuminuria range [23]. Among Type 2 Caucasian patients, the association of diabetes duration and microalbuminuria is equivocal [35,36]. In the current study, patients with abnormal AER had longer known diabetes duration than those with normal AER (4.2 versus 3 years), for Type 1 and (7.5 versus 3 years, p < 0.001) for Type 2. On regression analysis, known diabetes duration was identified as a strong predictor for development of abnormal albuminuria in Type 2 patients.

Poor glycaemic control is well defined contributor to the development and progression of microalbuminuria among Type 2 patients [35-38], as well as Type 1 patients [22,39,40]. In contrast, most of the available studies from sub-Saharan African including the present study fail to demonstrate any significant relationship between the level of glycaemic control and microalbuminuria. Goldschmid et al demonstrated a high prevalence of microalbuminuria and nephropathy among African-American diabetic patients, they also showed that metabolic control was not associated with the disease [7].

Elevated blood pressure is documented as the most significant contributing factor in the pathogenesis and the progression of abnormal AER and eventually development of diabetic nephropathy in both Type 1 and Type 2 diabetic patients [41-43]. In the present study, the median SBP and DBP were lower among Type 1 than among Type 2 patients. When SBP and DBP of Type 1 patients with normal AER were compared with those with abnormal AER there was no significant differences. In contrast however, Type 2 patients with abnormal AER had statistically significant higher SBP and DBP than those with normal AER, (p < 0.0001).

The number of Type 1 diabetic patients in this study was limited and few had microalbuminuria. These limitations probably account for the lack of statistical correlation to the commonly acknowledged risk factors for microalbuminuria. Other limitations of the current study include those inherent to patient reliability or compliance in complete urine collection and recording correctly the timed urine collections. Incomplete urine collection will have an effect of lowering the calculated AER and eventually underestimating the actual prevalence of microalbuminuria. This is however not supported by the calculated ACR from the same sample that showed an agreement between ACR and AER, the kappa coefficiency was 0.85, 95%CI of (0.76, 0.95) which is considered to be a very good level of agreement.

## Conclusion

The occurrences of microalbuminuria detected in this study are much lower than previously reported in studies from sub-Saharan Africa and similar to what have been found in Caucasian diabetic patients. In Type 2 diabetic patients the duration of diabetes was the strongest predictor; SBP and serum creatinine as well predicted increased albumin excretion rate.

## Competing interests

The author(s) declare that they have no competing interests.

## Authors' contributions

JJKL the principal investigator participated in the study design, data collection and coordinated data management and analysis. Also wrote the initial and revisions of the manuscript. HT and KV participated in study design, data analysis and manuscript reviews and ZG participated in data collection and management. All authors read and approved the final manuscript.

## Pre-publication history

The pre-publication history for this paper can be accessed here:



## References

[B1] Raine AE (1993). Epidemiology, development and treatment of end-stage renal failure in type 2 (non-insulin-dependent) diabetic patients in Europe. Diabetologia.

[B2] Ritz E (1999). Nephropathy in type 2 diabetes. J Intern Med.

[B3] Ballard DJ, Humphrey LL, Melton LJ, Frohnert PP, Chu PC, O'Fallon WM, Palumbo PJ (1988). Epidemiology of persistent proteinuria in type II diabetes mellitus. Population-based study in Rochester, Minnesota. Diabetes.

[B4] Hasslacher C, Ritz E, Wahl P, Michael C (1989). Similar risks of nephropathy in patients with type I or type II diabetes mellitus. Nephrol Dial Transplant.

[B5] Orchard TJ, Dorman JS, Maser RE, Becker DJ, Drash AL, Ellis D, LaPorte RE, Kuller LH (1990). Prevalence of complications in IDDM by sex and duration. Pittsburgh Epidemiology of Diabetes Complications Study II. Diabetes.

[B6] Parving HH, Gall MA, Skott P, Jorgensen HE, Lokkegaard H, Jorgensen F, Nielsen B, Larsen S (1992). Prevalence and causes of albuminuria in non-insulin-dependent diabetic patients. Kidney Int.

[B7] Goldschmid MG, Domin WS, Ziemer DC, Gallina DL, Phillips LS (1995). Diabetes in urban African-Americans. II. High prevalence of microalbuminuria and nephropathy in African-Americans with diabetes. Diabetes Care.

[B8] Crook ED (2002). Diabetic renal disease in African Americans. Am J Med Sci.

[B9] Rahlenbeck SI, Gebre-Yohannes A (1997). Prevalence and epidemiology of micro- and macroalbuminuria in Ethiopian diabetic patients. J Diabetes Complications.

[B10] Sobngwi E, Mbanya JC, Moukouri EN, Ngu KB (1999). Microalbuminuria and retinopathy in a diabetic population of Cameroon. Diabetes Res Clin Pract.

[B11] Erasmus RT, Oyeyinka G, Arije A (1992). Microalbuminuria in non-insulin-dependent (type 2) Nigerian diabetics: relation to glycaemic control, blood pressure and retinopathy. Postgrad Med J.

[B12] Wanjohi FW, Otieno FCF, Ogola EN, and Amayo EO (2002). Nephropathy in patients with recently diagnosed Type 2 diabetes mellitus in Black Africas. East African Medical Journal.

[B13] Hasslacher C, Bostedt-Kiesel A, Kempe HP, Wahl P (1993). Effect of metabolic factors and blood pressure on kidney function in proteinuric type 2 (non-insulin-dependent) diabetic patients. Diabetologia.

[B14] Mogensen CE (1987). Microalbuminuria as a predictor of clinical diabetic nephropathy. Kidney Int.

[B15] Alberti KG, Zimmet PZ (1998). Definition, diagnosis and classification of diabetes mellitus and its complications. Part 1: diagnosis and classification of diabetes mellitus provisional report of a WHO consultation. Diabet Med.

[B16] WHO (1999). International Society of Hypertension guidelines for the manegement of hypertension.. J Hypertens.

[B17] Hiar CE (1998). Clinical summary report. Clinical performance of DCA 2000+ hemoglobin A1c system six-minute assay.. Bayer Diagnostic Division 1-7, April.

[B18] (2005). The Diabetes Research in Children Network (DirectNet) Study Group. Comparison of fingerstick hemoglobin A1c levels assayed by DCA2000 with the DCCT/EDIC central laboratory assay: results of a Diabetes Research in Children Network (DirectNet) Study. Pediatric Diabetes.

[B19] Cockcroft DW (1976). Gault MH. Prediction of creatinine clearance from serum creatinine. Nephron.

[B20] Levey AS, Coresh J, Balk E, Kausz AT, Levin A, Steffes MW, Hogg RJ, Perrone RD, Lau J, Eknoyan G (2003). National Kidney Foundation practice guidelines for chronic kidney disease: evaluation, classification, and stratification. Ann Intern Med.

[B21] (1999). Predictors of the development of microalbuminuria in patients with Type 1 diabetes mellitus: a seven-year prospective study. The Microalbuminuria Collaborative Study Group. Diabet Med.

[B22] Cederholm J, Eliasson B, Nilsson PM, Weiss L, Gudbjornsdottir S (2005). Microalbuminuria and risk factors in type 1 and type 2 diabetic patients. Diabetes Res Clin Pract.

[B23] Marshall SM, Alberti KG (1989). Comparison of the prevalence and associated features of abnormal albumin excretion in insulin-dependent and non-insulin-dependent diabetes. Q J Med.

[B24] Mathiesen ER, Saurbrey N, Hommel E, Parving HH (1986). Prevalence of microalbuminuria in children with type 1 (insulin-dependent) diabetes mellitus. Diabetologia.

[B25] Bruno G, Cavallo-Perin P, Bargero G, Borra M, Calvi V, D'Errico N, Deambrogio P, Pagano G (1996). Prevalence and risk factors for micro- and macroalbuminuria in an Italian population-based cohort of NIDDM subjects. Diabetes Care.

[B26] Gatling W, Mullee MA, Hill RD (1988). The prevalence of proteinuria detected by Albustix in a defined diabetic population. Diabet Med.

[B27] Gall MA, Rossing P, Skott P, Damsbo P, Vaag A, Bech K, Dejgaard A, Lauritzen M, Lauritzen E, Hougaard P (1991). Prevalence of micro- and macroalbuminuria, arterial hypertension, retinopathy and large vessel disease in European type 2 (non-insulin-dependent) diabetic patients. Diabetologia.

[B28] Collins AC, Sethi M, MacDonald FA, Brown D, Viberti GC (1993). Storage temperature and differing methods of sample preparation in the measurement of urinary albumin. Diabetologia.

[B29] Osberg I, Chase HP, Garg SK, DeAndrea A, Harris S, Hamilton R, Marshall G (1990). Effects of storage time and temperature on measurement of small concentrations of albumin in urine. Clin Chem.

[B30] Hara F, Nakazato K, Shiba K, Shimoda J, Kojima T, Fukumura Y, Kobayashi I (1994). Studies of diabetic nephropathy. I. Effects of storage time and temperature on microalbuminuria. Biol Pharm Bull.

[B31] Hovind P, Tarnow L, Rossing K, Rossing P, Eising S, Larsen N, Binder C, Parving HH (2003). Decreasing incidence of severe diabetic microangiopathy in type 1 diabetes. Diabetes Care.

[B32] Mohsin F, Craig ME, Cusumano J, Chan AK, Hing S, Lee JW, Silink M, Howard NJ, Donaghue KC (2005). Discordant trends in microvascular complications in adolescents with type 1 diabetes from 1990 to 2002. Diabetes Care.

[B33] Viberti G (1988). Etiology and prognostic significance of albuminuria in diabetes. Diabetes Care.

[B34] (1992). Microalbuminuria in type I diabetic patients. Prevalence and clinical characteristics. Microalbuminuria Collaborative Study Group. Diabetes Care.

[B35] Schmitz A, Vaeth M (1988). Microalbuminuria: a major risk factor in non-insulin-dependent diabetes. A 10-year follow-up study of 503 patients. Diabet Med.

[B36] Gall MA, Hougaard P, Borch-Johnsen K, Parving HH (1997). Risk factors for development of incipient and overt diabetic nephropathy in patients with non-insulin dependent diabetes mellitus: prospective, observational study. Bmj.

[B37] (1998). Tight blood pressure control and risk of macrovascular and microvascular complications in type 2 diabetes: UKPDS 38. UK Prospective Diabetes Study Group. Bmj.

[B38] Phillips CA, Molitch ME (2002). The relationship between glucose control and the development and progression of diabetic nephropathy. Curr Diab Rep.

[B39] (1995). Effect of intensive therapy on the development and progression of diabetic nephropathy in the Diabetes Control and Complications Trial. The Diabetes Control and Complications (DCCT) Research Group. Kidney Int.

[B40] Wiseman M, Viberti G, Mackintosh D, Jarrett RJ, Keen H (1984). Glycaemia, arterial pressure and micro-albuminuria in type 1 (insulin-dependent) diabetes mellitus. Diabetologia.

[B41] Mogensen CE (2003). Microalbuminuria and hypertension with focus on type 1 and type 2 diabetes. J Intern Med.

[B42] Schmitz A, Vaeth M, Mogensen CE (1994). Systolic blood pressure relates to the rate of progression of albuminuria in NIDDM. Diabetologia.

[B43] (1993). UK Prospective Diabetes Study (UKPDS). X. Urinary albumin excretion over 3 years in diet-treated type 2, (non-insulin-dependent) diabetic patients, and association with hypertension, hyperglycaemia and hypertriglyceridaemia. Diabetologia.

